# Comparing Text-Based and Virtual Reality–Based Approaches for AI-Enhanced Instruction in Functional Communication Training: Exploratory Randomized Trial

**DOI:** 10.2196/105569

**Published:** 2026-10-29

**Authors:** Seth Andrew King, Lee Flores, Eunhae Cho, May Kannika Ross, Collin Glazek, Casey Coghlan, Tyler Bell

**Affiliations:** 1 Department of Teaching and Learning, College of Education, University of Iowa, 240 S Madison St, Iowa City, IA, 52242, United States, 1 3193355324; 2 University of Iowa, Iowa City, IA, United States; 3 Department of School Psychology, College of Education, University of Iowa, Iowa City, IA, United States; 4 Electrical and Computer Engineering, College of Engineering, University of Iowa, Iowa City, IA, United States

**Keywords:** virtual reality, artificial intelligence, computer-assisted instruction, educator training, generalization

## Abstract

**Background:**

Training practitioners to implement functional communication training (FCT) is resource intensive. Emerging technologies, such as virtual reality (VR) and chatbots, may deliver training at scale. Their comparative effectiveness and whether skills taught in one modality transfer to another remain uncertain.

**Objective:**

This exploratory study evaluated the effect of multicomponent training packages featuring either VR or a chatbot on (1) posttraining performance on FCT implementation probes administered in both formats and (2) changes in participants’ self-perceived knowledge, confidence, and usefulness.

**Methods:**

A convenience sample of 23 university students (mean age=24 y; 15/23, 65% female) were recruited through classroom announcements and peer referrals. Eligible participants (at least 18 years of age, available to attend trainings, and who had no history of discomfort associated with VR) were randomly assigned to either VR (n=12) or chatbot (n=11) training. Groups did not differ significantly across demographic characteristics. In-person trainings consisted of video-recorded instruction and rehearsal of steps in VR or with the chatbot during a single session. Approximate times of sessions, including tutorials and assessments, were 45 minutes (VR) and 55 minutes (chatbot). All participants’ performance on FCT procedures was measured using 2 formats: a VR-based observed mastery probe (OMP) and a text-based probe (TBP). Participants also completed a survey measuring their self-perceptions.

**Results:**

The number of FCT steps participants performed correctly (of 18) was analyzed using a binomial generalized linear mixed model, with random intercepts for participant and fixed effects of training group (chatbot vs VR), time (pretest vs posttest), and probe modality (OMP vs TBP); *α*=.05. Results indicated a main effect of time (odds ratio [OR] 36.09, 95% CI 20.75‐62.75; *P*<.001). A significant 3-way interaction (time × probe modality × training group) indicated that the pattern of skill transfer differed by training condition and probe type (OR 6.10, 95% CI 2.08‐17.86; *P<*.001). At posttest, the chatbot group significantly outperformed the VR group on the TBP (OR 4.14, 95% CI 1.20‐14.24; *P=*.049), completing, on average, 4 more steps in the procedure (average marginal effect 0.22, 95% CI 0.04‐0.41). Differences between groups on the OMP were not significant (OR 0.61, 95% CI 0.18‐2.07; *P*=.43). Across all self-report measures, there was no differential improvement by training condition at posttest.

**Conclusions:**

This study provides the first comparison of chatbot- and VR-based training for FCT. This extends prior work that compared VR against didactic instruction or assessed performance using a single modality. Findings contribute to the field by supporting the viability of text-based chatbots in training and underscoring the importance of the alignment between training and assessment modalities. Chatbot-enhanced training systems may extend access to evidence-based techniques, such as FCT, in areas with limited training resources. More work is needed to confirm effects and demonstrate applicability to practice.

## Introduction

### Problem

Self-injurious behavior and aggression manifest in as many as 30% of individuals with intellectual and developmental disabilities (IDD) [1]. Beyond physical harm, these behaviors produce chronic stress for families, economic strain on service systems, and overall disruptions to stakeholder well-being [2]. Restrictive placement, chemical/physical restraints, and punishment often used to suppress severe challenging behaviors are not long-term solutions and are associated with negative outcomes [3]. Educators and other personnel in service settings require training in nonpunitive, effective methods for reducing challenging behaviors and achieving optimal outcomes for people with IDD [4].

Challenging behaviors have many potential causes [5]. Functional communication training (FCT) is one of the most widely validated interventions for reducing aggression and self-injury associated with social causes in children with IDD by teaching appropriate communication alternatives (ie, replacement behaviors, [6,7]). Instead of hitting adults to escape from difficult tasks, for example, children can be taught to request assistance (eg, “I don’t understand,” [8]). FCT has been shown to be effective at reducing challenging behaviors, often yielding improvements in behavior and communication [6,9].

Despite its demonstrated effectiveness, the dissemination of FCT may be hindered by the complexity of the procedure and intensive nature of staff preparation [10]. Effective training typically involves didactic instruction, modeling, rehearsal with feedback, and in-situ performance checks, all of which require expert trainers and access to clients or trained confederates [10,11]. These logistical and resource-intensive demands pose challenges to scaling FCT, especially when opportunities for rehearsal are limited. Recent reviews note that, although effective training improves implementation of FCT, studies vary widely in their use of role-play, simulations, and direct client application [10]. Such constraints can impede skill fluency and may slow adoption in schools and settings facing shortages in personnel or other resources.

### Review of Relevant Scholarship

Advancements in digital training have increasingly positioned immersive virtual reality (VR), or interfaces (eg, head-mounted displays, motion sensors) that situate participants within virtual space [12], as a promising modality for training educators and service providers (eg, [13,14]). For the purposes of training individuals to use FCT, VR places trainees inside realistic, 3-dimensional scenarios populated by avatars that emit challenging behavior, with feedback adjusted based on the trainee’s performance. Clay and colleagues [13] demonstrated the feasibility of VR-based opportunities for practice among small cohorts (n=6‐13) of novice graduate students and clinicians [15]. Training consisted of lecture, modeling the technique within VR, prompting, and feedback. Trainees demonstrated mastery of the procedure by engaging with a scripted avatar within the simulation. Across studies, some trainees acquired the procedure without proceeding through the entire training [13]; others required additional training from clinicians following VR rehearsal [15]. Gains from VR training transferred to brief live role-play simulations [15].

One barrier to the use of VR simulations for training is the need for professional oversight; VR provides opportunities to rehearse a wide range of skills but, on its own, does not alleviate shortages of qualified supervisors in disability service fields. Artificial intelligence (AI), broadly defined as software capable of acquiring, processing, and acting upon information [16], has the potential to reduce the need for human assistance for tasks such as assessment and feedback in VR simulations [17]. Generative AI and natural language processing can create speech for avatars and assess written or spoken trainee responses; however, much of this work involves speech or text-based interaction, with limited emphasis on the behaviors often addressed through FCT [10].

King and colleagues [18] evaluated an AI-augmented FCT simulation in VR using an automated training model incorporating speech-to-text, text-to-speech, text classification, and motion sensing. In 2 small, single-case design studies, participants received prerecorded instruction, video models, and automated systematic prompting, with performance measured through in-simulation speech and motion tracking. Trainees exhibited improved FCT skills within the simulation following training. A subsequent randomized controlled trial [19] evaluated the simulation with 49 general education undergraduates using a waitlisted design, randomly assigning participants to either an intervention group receiving lecture, modeling, and VR practice or a control group receiving lecture and modeling only. Results indicated that the VR group demonstrated significantly more correct FCT steps at posttest (*d*=2.45) and maintenance (*d*=1.58) compared with controls, with correspondence between AI-administered and human-observed assessments averaging approximately 90%, though groups did not differ on content knowledge tests or self-reported confidence and assessments were conducted exclusively within the simulation environment.

Despite these promising findings, VR remains less attractive as an instructional platform due to concerns regarding the cost of hardware and its acceptability across diverse learners [19]. Trainee motions that the system acknowledges, as well as characteristics of the avatar and virtual world, must be programmed into the lesson in advance. Thus, opportunities to provide trainees with multiple exemplars of practical situations are limited. The cumbersome nature of VR, which can be difficult to implement in crowded spaces and difficult to use for individuals without previous experience, represents an additional impediment to its application in many training settings. Additionally, there are critical issues that undermine positive findings of many VR training studies [20]. One of the most persistent involves the frequent misalignment between intervention conditions featuring VR and control conditions, which often differ in terms of both opportunities for practice and other crucial elements of instruction [21]. The imbalance impedes attempts to isolate the unique advantages of immersive training. However, comparing AI-augmented VR to high-quality training featuring multiple opportunities to rehearse practice may also be inappropriate, as these systems are often intended to compensate for resource constraints that prevent optimal training [19].

Chatbots—or computer-based applications that can engage humans in conversations through spoken or written text—represent an increasingly popular AI tool in higher education settings [22]. Uses for chatbots range from the generation of learning exercises, planning documents, conversation partners, and assistance in writing assignments [23,24]. Role-play activities designed to assist in the acquisition of procedural skills, such as those facilitated by VR, are relatively scarce in the chatbot literature [22]. Nonetheless, the studies that have examined this topic suggest that chatbots designed to engage students in role-play might improve procedural accuracy and fluency (eg, [25]). Compared with VR, chatbots may be able to provide a wider range of learning scenarios and, when coupled with large language models (LLMs; eg, ChatGPT), may be able to provide rich feedback in relation to targeted skills. On the other hand, King and colleagues [26] suggested that immersion and opportunities to rehearse relevant physical activities might make VR more suited to learning complex procedures such as FCT. Given the relative ease at which chatbots may be deployed, the extent to which skills learned in a purely textual format generalize to physical activities as well as the utility of chatbots in learning steps in the procedure more generally warrant further examination.

Chatbots can process learner input, provide adaptive feedback, and simulate interactive dialogue in a manner similar to supports featured in previous instructional VR studies (eg, [19]). In educational contexts, chatbots are increasingly regarded as scalable tools to support personalized learning and address the diverse needs of classrooms [23]. However, text-based formats do not simulate aural and visual stimuli encountered in authentic environments or require the learner to engage in physical or spoken behaviors associated with appropriate practice. These features are associated with effective training in behavioral service fields [27] and represent one of the purported advantages of the VR format [28], particularly in the context of behavior analytic theories of learning emphasizing correspondence between the modalities engaged during learning and performance [29-31]. More specifically, instruction and assessment can be characterized as learning channels that represent both how stimuli are perceived (eg, see) and how a response is produced (eg, say) [29,30]. The primary implication is that instruction must explicitly support this relationship, particularly if the learner does not have experience with the relevant channels [31].

Cognitive load theory, which proposes that humans have limited mental capacity for processing information and storing it in long-term memory [32], has further implications for training simulation. Intrinsic cognitive load refers to the mental demands associated with the complexity of the information and interactions relevant to instructional goals (eg, breadth of information required for adequate performance); extraneous load refers to additional mental resources expended in processing stimuli irrelevant to instructional goals (eg, reconciliation of various media used in instruction) [33]. Although this would appear to support the deployment of chatbot simulations or simulations that do not require management of controllers or other materials [34], VR may be better positioned to fill the gap between text and the physical and mental demands of practice [13,15,18]. Recent research further suggests greater task complexity can be tolerated by trainees [35] and may promote deeper learning [36].

Research on chatbot-based training for preservice teachers, and especially for teaching FCT, remains limited. No published studies have directly compared VR-based FCT training simulations to text-based alternatives, leaving questions about their relative effectiveness and generalization. Additionally, a primarily text-based medium may not be suitable for teaching skills comprising both speech and physical action, which has motivated much of the VR scholarship in this area [13,15,18]. This gap highlights the need for systematic evaluation of chatbot training alongside VR in the context of FCT.

### Aims and Objectives

This exploratory study addresses questions regarding both the relative advantages of VR-based training compared with aligned alternatives and the viability of chatbots as tools for teaching instructional procedures. Building on two recent works that established the technological infrastructure and preliminary empirical support for automated VR–based behavioral skills training in FCT [18,19], we examined performance within and across VR and chatbot training modalities to examine whether skills acquired in one modality generalize effectively to the other. By investigating these aspects, this study explored the potential of various instructional modalities for training individuals in evidence-based behavioral interventions. The study was guided by the following primary question: To what extent does improved performance from pre- to posttraining differ by training modality and probe format? A secondary question pertained to participant perceptions of learning following the intervention: To what extent do participants’ perceived knowledge, confidence, and utility of the procedure vary based on training modality?

## Methods

### Ethical Considerations

All study procedures and consent forms were approved by the institutional review board (IRB) of a midwestern public university in the United States as exempt prior to the commencement of participant recruitment (2022409364). Although all procedures were disclosed to the IRB, this study was not registered. The first author met with interested students via teleconferencing software to describe the study in greater detail and confirm participation. Written consent was obtained prior to the initiation of procedures. Study exclusion criteria were developed to prevent discomfort or exacerbation of conditions associated with the use of VR. Participants were advised to terminate sessions if they experienced discomfort; no attrition or adverse events occurred, however. All initial data were confidential, with numbers used in the place of names; all codes linking individuals to data were destroyed after the conclusion of the study. Text transmitted to OpenAI during chatbot sessions consisted of typed statements, described actions, and other procedurally constrained responses directed at a simulated student within partially scripted scenarios. No names, codes, or identifying information were disclosed. Data-sharing options in the OpenAI platform were disabled across all settings for model feedback, evaluation, inputs, and outputs. Consent procedures therefore addressed confidentiality and chatbot participation without specifically itemizing data transmission. No figures or supplementary materials permit the identification of participants. Secure digital media were used for data storage; paper forms were secured in a locked office. A US $10 gift card was offered as an incentive for participation. All participants accepted payment upon conclusion of the study. This study is reported in accordance with CONSORT-EHEALTH (Consolidated Standards of Reporting Trials of Electronic and Mobile Health Applications and Online Telehealth) [37].

### Inclusion and Exclusion

Eligibility criteria included being at least 18 years old, a currently enrolled student at the university in which the study was conducted, proficient in English, having no history of epilepsy or discomfort associated with virtual reality usage, and availability for on-campus meetings scheduled in March 2025 and April 2025.

### Participant Characteristics

A total of 23 participants voluntarily enrolled in the study. Participants ranged in age from 19 years to 42 years (mean 24.0, SD 5.3 years). Of the participants, 15 identified as female, 7 identified as male, and 1 did not disclose. Most (21/23, 91%) were undergraduates, with an average of 2.8 (SD 1; range=1‐5) years in their respective programs including education, special education, and fields outside of education. Demographic surveys indicated there were no significant differences in participant characteristics or familiarity with VR or AI assistants prior to the study. We assigned participants to either the VR training (n*=*12) or chatbot training (n*=*11) groups.

### Sampling Procedures

Recruitment, which extended from January 2025 to March 2025, was conducted through convenience sampling with a snowball component. Specific methods included (1) in-class announcements by the instructor of a classroom management course with a total enrollment of 90 and (2) peer referrals among students enrolled in computer engineering courses. Participation through both channels was determined via self-selection. Ten students from the classroom management course participated, resulting in a participation rate of 11% (10/90). Given the nature of snowball sampling, it was not possible to determine a recruitment rate for students from engineering courses.

The project was conducted over 2 weeks in April 2025. Each participant completed 1 session per week across 2 consecutive weeks, with sessions scheduled to ensure approximately 7 days elapsed between sessions. For each session, the first author scheduled individual, face-to-face meetings at a time of convenience for participants. Sessions were conducted individually in an office area equipped with reliable internet access and the necessary equipment.

### Sample Size, Power, and Precision

No a priori power analysis was conducted. To our knowledge, no published study had compared chatbot and VR training in the current instructional model. Consequently, no effect size was available to derive a target sample. No target sample size was set in advance, and no interim analyses nor stopping rules were used. Enrollment was constrained by the limited number of people who responded through recruitment channels, in addition to restricted availability of personnel and funds beyond the study window. All recruited participants successfully completed the study. Precision of parameter estimates of the exploratory analyses was determined using 95% confidence intervals.

### Measures and Covariates

Primary outcomes included the observed mastery probe (OMP) and the text-based probe (TBP). Secondary outcomes related to participants’ perceptions and experiences were obtained through responses to the SKIL Survey [38]. No covariates were included. A social validity survey assessing acceptability of the intervention was also administered. Participants completed a demographic survey used for describing the sample and assessing equivalence between groups.

#### Primary Measures

##### Observed Mastery Probe

The OMP measured the percentage of steps, including statements and gestures, of an FCT procedure completed correctly by participants while interacting with a child avatar in the simulation [18,19]. Scenarios required the exhibition of specific responses in relation to a simulated child whose headbanging or tantrums were linked to accessing a preferred item or avoiding an academic task. Replacement behaviors could include vocal requests (eg, “I want a break”), use of hand gestures, or a response card (ie, touching a card corresponding with a request; eg, [39,40]). Researchers provided a scripted description of the challenging behavior, its function, and the proposed behavior intervention (ie, encourage the child to use a replacement behavior). The sequence and content of probes differed from those featured in VR training sessions to reduce the possibility of memorization. The precise content of each OMP appears in King et al [19]. We calculated results by dividing the number of steps correctly exhibited by the total number of steps in each lesson (ie, 18 steps).

##### Text-Based Probe

The TBP measured the percentage of typed responses correctly corresponding with the steps of an FCT procedure participants exhibited while interacting with a simulated child using a text-based messaging interface. Scenarios represented series of text passages designed to correspond with individual instances that occurred in the VR simulation (eg, “Student begins pounding their hands on a desk; Student says ‘Can I have a break’”). As with the OMP, researchers described the context of the situation (eg, challenging behavior) before initiating the TBP. Participants responded to the simulated child by typing messages, which were exchanged in a back-and-forth format. Messages describing actions and statements of the simulated child were generated by an LLM (GPT-4o) configured to role-play within each scenario. The sequence and content of probes differed from those featured in chatbot training sessions to reduce the possibility of memorization. The order of steps matched those featured in the OMP. Scores were calculated in accordance with methods used for the OMP.

### Secondary Measures

#### SKIL Survey

The SKIL Survey was used to assess participants’ self-reported knowledge, confidence, and perceived usefulness of concepts relevant to training. This survey has a 4-point Likert-type scale ranging from 1 (none/very low) to 4 (substantial/very high). Participants rated 9 items across all scales (eg, functional assessment-based intervention).

#### Social Validity

A survey adapted from previous studies (eg, [19,41]) was used to assess the acceptability of the simulation to participants. The survey assessed 4 domains: acceptability (the extent to which participants found the intervention agreeable and appropriate), effectiveness (perceived improvement in relevant skills), VR (ease of use of the VR system), and chatbot (ease of use of the chatbot system). Participants responded to statements concerning the project (eg, “The use of VR was acceptable to me”*; “*I had no difficulty using VR”) using a 6-point scale (1=strongly disagree; 6=strongly agree). Participants also answered a series of open-ended questions related to their experiences in the simulation.

### Data Collection

OMP data were collected through direct observation of participants’ use of VR at baseline and training sessions. All OMPs were terminated by observers as soon as participants exhibited an incorrect response or failed to respond within 4 seconds (ie, single-opportunity method; [42]). Steps participants did not complete due to termination were counted as incorrect. Feedback was not provided following OMPs.

As with the OMP, researchers observing typed responses from a secondary screen assessed responses during the baseline and training sessions. TBPs were terminated immediately following an incorrect response. Participants who did not initiate typing after 4 seconds were reminded that they could opt out at any time. A time-based discontinuation rule was not observed due to unpredictable duration of typed responses relative to actions or brief statements required during OMPs. However, the interval between each simulated student message and participants’ submission of responses—which includes the duration needed to read, compose, and type the response—was logged by the system. Participants did not receive feedback during TBPs.

Participants completed the SKIL Survey during baseline and the training sessions. Participants accessed a digital version of the SKIL Survey using a Qualtrics link. A digital social validity survey was completed at the end of the training session.

### Quality of Measurements

Interobserver agreement (IOA) was collected for OMP and TBP across multiple phases of the project. The first author trained observers prior to the project by scoring videos depicting nonparticipants. Observers were required to master the training criteria of scoring 3 consecutive videos with greater than 90% agreement with the first author before finishing training. Agreement was defined as 2 observers independently recording an identical participant response for the same step of the procedure. Scheduling allowed IOA collection for 87% (20/23) of all probes administered within baseline and 61% (14/23) of all intervention session probes across VR and chatbot groups (ie, VR=58.33% and chatbot=63.64%).

### Instrumentation

The OMP was created by a multidisciplinary team including engineers, behavior analysts, and school psychologists and subsequently evaluated by a panel of experts in behavior intervention [18,19]. The TBP was directly modeled after the OMP by the original development team. The SKIL Survey is commonly featured in personnel preparation studies involving behavior intervention [43,44]. Adapted SKIL Surveys have demonstrated acceptable Cronbach α for knowledge (0.91), confidence (0.88), and usefulness (0.92) at baseline (eg, [43]). The social validity survey was adapted from previous VR studies [19,41].

### Psychometrics

IOA was calculated by dividing the number of agreements by the total number of steps observed. Average IOA for baseline sessions was 100% for OMP and 98.75% (SD 5.59%; range=75%‐100%) for TBP. The average training session IOA for the VR group was 92.21% (SD 12.01%; range=66.67%‐100%) for the OMP and 94.29% (SD 12.42%; range=66.67%‐100%) for the TBP. For the chatbot group, the average IOA for the OMP and TBP was 100%. Disagreements were reconciled via consensus, and reconciled scores were subsequently analyzed. For the SKIL Survey, Cronbach α for all subscales among the current sample was acceptable (knowledge, 0.95; confidence, 0.90; usefulness, 0.92) at baseline.

### Conditions and Design

Conditions were experimentally manipulated. We analyzed the effectiveness of the 2 multicomponent training packages using an independent measures pretest-posttest design with 2 conditions: the VR condition (ie, lecture, model, and VR practice) or a chatbot condition (ie, lecture, model, and chatbot practice). All participants completed both the OMP and TBP at baseline and following training regardless of assigned condition.

#### Random Assignment Method

Individual participants were the unit of randomization. The first author enrolled and randomly assigned participants using a stratified randomization procedure [45] based on the results of the OMP and TBP (see the Measures and Covariates section) following the baseline session. Participants who scored >11% of items correctly on either the VR or text probe were assigned separately. Randomization was achieved using Excel (Microsoft Corp).

#### Random Assignment Implementation and Concealment

Participants were randomly assigned codes following enrollment. The first author assigned participants without reference to participant identities using a file containing only codes and baseline probe scores. Participants were unaware of their assigned condition until after baseline assessment and randomization were complete.

#### Masking

Masking was not feasible in this study. Participants were never directly informed of their training assignment and used both the chatbot and VR in baseline and training regardless of assignment. It is likely, however, that participants knew which of the 2 technologies was used for training. The first author, who administered all sessions, was likewise aware of condition assignments, as were observers scoring OMP and TBP. Scorer influence was nonetheless constrained by the scoring of probes against a fixed task analysis and collection of IOA across a high proportion of sessions.

#### Experimental Manipulations

##### Baseline

During the baseline session, researchers collected demographic information and administered custom tutorials involving the use of the VR and chatbot systems unrelated to FCT. For the VR tutorial, participants received a brief, scripted description of the headset and motion controls from the researchers. Participants then completed a series of actions in the virtual space, including activating the microphone to speak, raising a hand until acknowledged by the simulation, moving a small cube, placing a hand on a specified area, and holding the student avatar’s hand. A full description of the tutorial appears in King et al [19]. The chatbot tutorial addressed similar content (eg, hand raising) but emphasized (1) using different regions of the screen for message exchanges or feedback and (2) using asterisks to denote physical actions. Following the tutorials, participants completed OMP and TBP probes with accompanying descriptions (see the Measures and Covariates section) in random order using VR and a laptop computer, respectively. Afterward, they completed the SKIL Survey. Average completion time for baseline sessions was approximately 30 minutes.

##### Training

Participants received the training session approximately 1 week after the baseline session. Regardless of group assignments, participants reviewed the VR and chatbot tutorials in random order. Each group also observed 2 randomly presented video-recorded lectures totaling approximately 8 minutes in length concerning challenging behavior maintained by escape from assignments and access to preferred objects. Models of FCT applications appeared in each video. Additional detail regarding video content appears in King et al [18].

The same instructional sequence followed each video in the participant’s assigned modality: (1) an error-free prompting probe, in which step-by-step hints appeared immediately before each component of the FCT procedure, and (2) two delayed prompting probes, during which prompts describing the correct response were withheld unless the participant made an error (both conditions) or hesitated for more than 4 seconds (VR only). For both conditions, prompts consisted of on-screen text describing the appropriate action. An example of a prompt in VR appears in Figure 1. In both conditions, participants were required to complete the prompted step correctly before proceeding through the simulation—steps that participants continued to complete incorrectly were prompted until the correct response was exhibited. In sum, training consisted of 1 individually administered session with the following components: 2 video-recorded instructional models, 2 error-free probes, and 4 delayed prompting probes delivered either within VR or via chatbot. Neither intervention was tailored nor modified during the study. All participants within each condition received the same instructional components, lesson sequence, and prompt structure.

**Figure 1.**
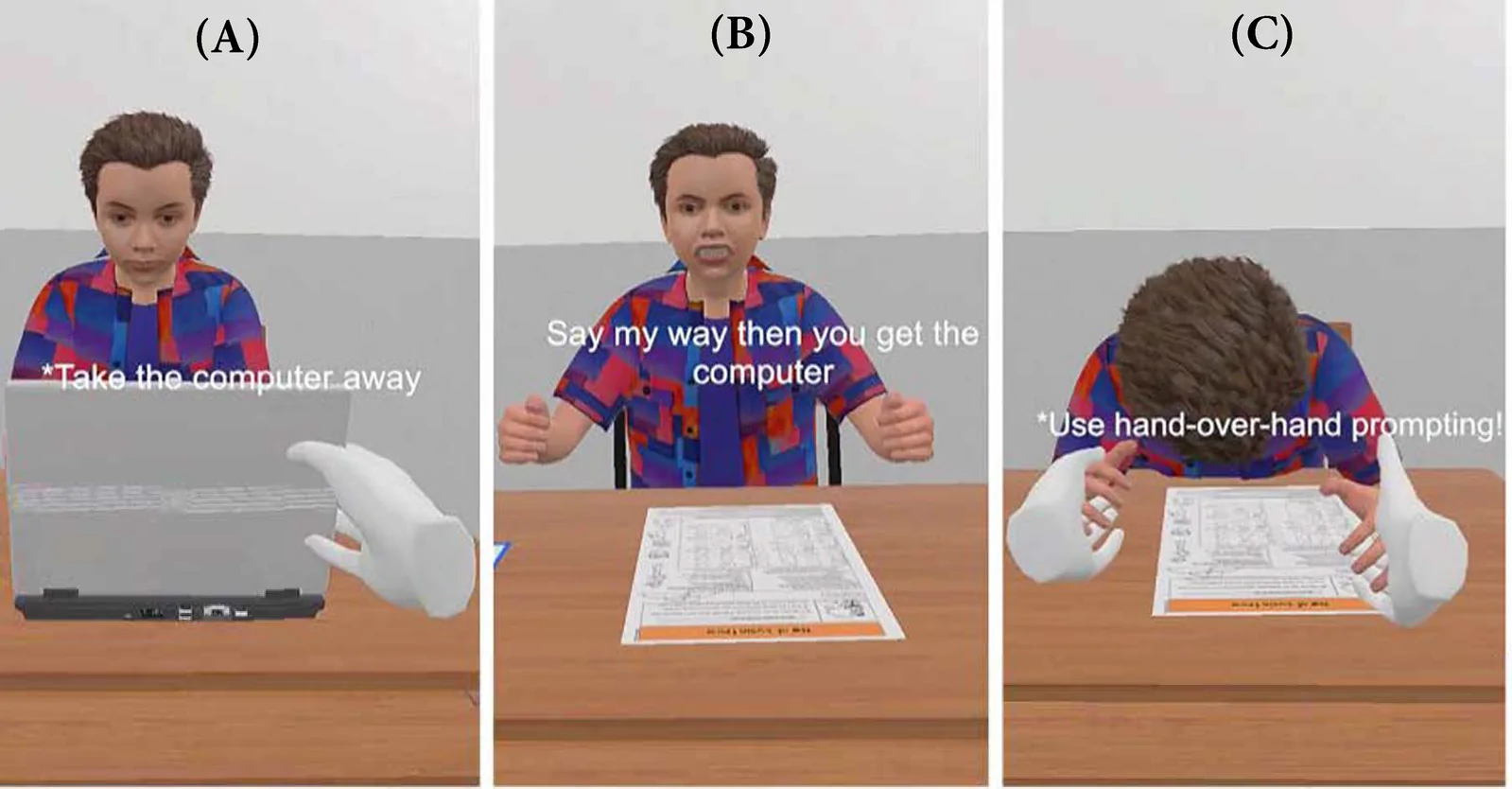
Depiction of interactions and on-screen prompts within the virtual reality (VR) simulation during training sessions in an exploratory randomized trial comparing chatbot- and VR-delivered instruction in functional communication training (FCT): (A) trigger for challenging behavior (removing the computer), (B) vocal prompt for communicative replacement behavior, and (C) physical guidance of a hand signal.

Across conditions, participants completed both the OMP and TBP. As in baseline, OMP and TBP were presented in random order. Sessions concluded with the administration of the SKIL and social validity surveys. Approximate durations of VR and chatbot group sessions including tutorials, training, and all completed assessments were 45 minutes and 55 minutes, respectively.

### Method of Experimental Manipulation Delivery

#### Virtual Reality

The VR simulation featured in this study extends a platform developed over several years by an interdisciplinary team of faculty in special education and computer engineering [18,19,41]. These simulations are predicated on behavioral theories of learning and instruction. A task analysis of FCT was arranged into branching pathways based on the range of responses a student might present (eg, target behavior, replacement behavior, compliance), with the resulting flowchart approved by experts in function-based intervention and used to derive individual lessons [46]. Developed in Unity, the simulation allowed trainees wearing a head-mounted display to interact with a virtual student in a simulated classroom. Trainees pressed a controller button to record vocal responses, which Google Cloud speech-to-text transcribed and Google’s Natural Language AI classified against phrases used to train the system; statements exceeding a confidence threshold of 0.75 were scored as correct. All in-situ AI scoring was immediately reviewed and approved by researchers before the simulation advanced. The simulation also assessed motion (eg, modeling a sign, physically guiding a response) using Unity’s motion-tracking features. A depiction of multiple sequences from the VR simulation appears in Figure 1. A complete description of the VR simulation, its development, and its assessment capabilities appear in King et al [18,19].

#### Chatbot

The text-based chatbot simulation was designed under the supervision of doctoral faculty in computer engineering to resemble the lessons and instructional logic of the VR simulation within a messaging interface. Student-teacher interactions were derived from the same flowcharts guiding the VR lessons, in which flowchart nodes represented a student action or a trainee response. Trainees interacted with the simulation on a laptop, selecting a lesson then responding to the simulated student through messages. A central panel displayed the exchange and a text field for trainee responses, while a side panel delivered prompts or feedback. Statements were typed without any special punctuation. Asterisks were used to denote physical actions described by the trainee.

The web interface was constructed in HTML, JavaScript, and CSS using the Svelte framework, with trainee data stored locally in a PostgreSQL database and lesson logic managed by a Flask (Python) backend. Lessons were implemented as fixed state sequences derived from the same FCT flowcharts used for VR. Identical lesson state sequences were used for all participants. Text generation was provided by OpenAI’s GPT-4o [47] through 2 distinct chatbot instances, one configured to role-play the simulated student and one to serve as an instructional assistant. The model was accessed through the OpenAI API using the “gpt-4o” identifier, which potentially represented successive versions of GPT-4o during data collection. Each instance was initialized with a system message specifying the classroom scenario, the student’s target behavior and its function, and the replacement behaviors being taught, along with instructions to remain in character and refrain from feedback regarding spelling or grammar. Instances were additionally initialized with a brief set of example exchanges from an unrelated instructional scenario that illustrated the required message structure and feedback format. The student instance generated target, replacement, and nonspecified behaviors appropriate to the scenario; the assistant instance generated feedback. Prompts differed across scenarios and target behaviors but were otherwise held constant. Each produced responses based on the accumulated context of the interaction. TBPs used the same state sequences without trainee-facing prompts. The student instance recorded student and trainee messages, whereas the assistant instance additionally recorded its own feedback, providing each chatbot with the context relevant to its role. Additional information regarding system prompts appears in Multimedia Appendix 1.

The model determined the surface wording of student responses but not their occurrence, content, or order. Each state specified both the instruction presented to the trainee (eg, “Verbally tell the student to complete the problems on a worksheet”) and a constraint on the simulated student’s response (eg, “the student engages in their target behavior”), which was rendered as text. Temperature, a hyperparameter that controls randomness and creativity of generative models ranging from 0.0 to 2.0, was maintained at the default 1.0 to preserve a balance of reproducibility and flexibility in LLM responses. No seed parameter was specified, allowing minor variation in wording across sessions within a fixed lesson structure. No generation failures were observed during data collection. As in VR, trainees passed a step when their response was approved by a researcher. A depiction of the chatbot interface appears in Figure 2.

**Figure 2.**
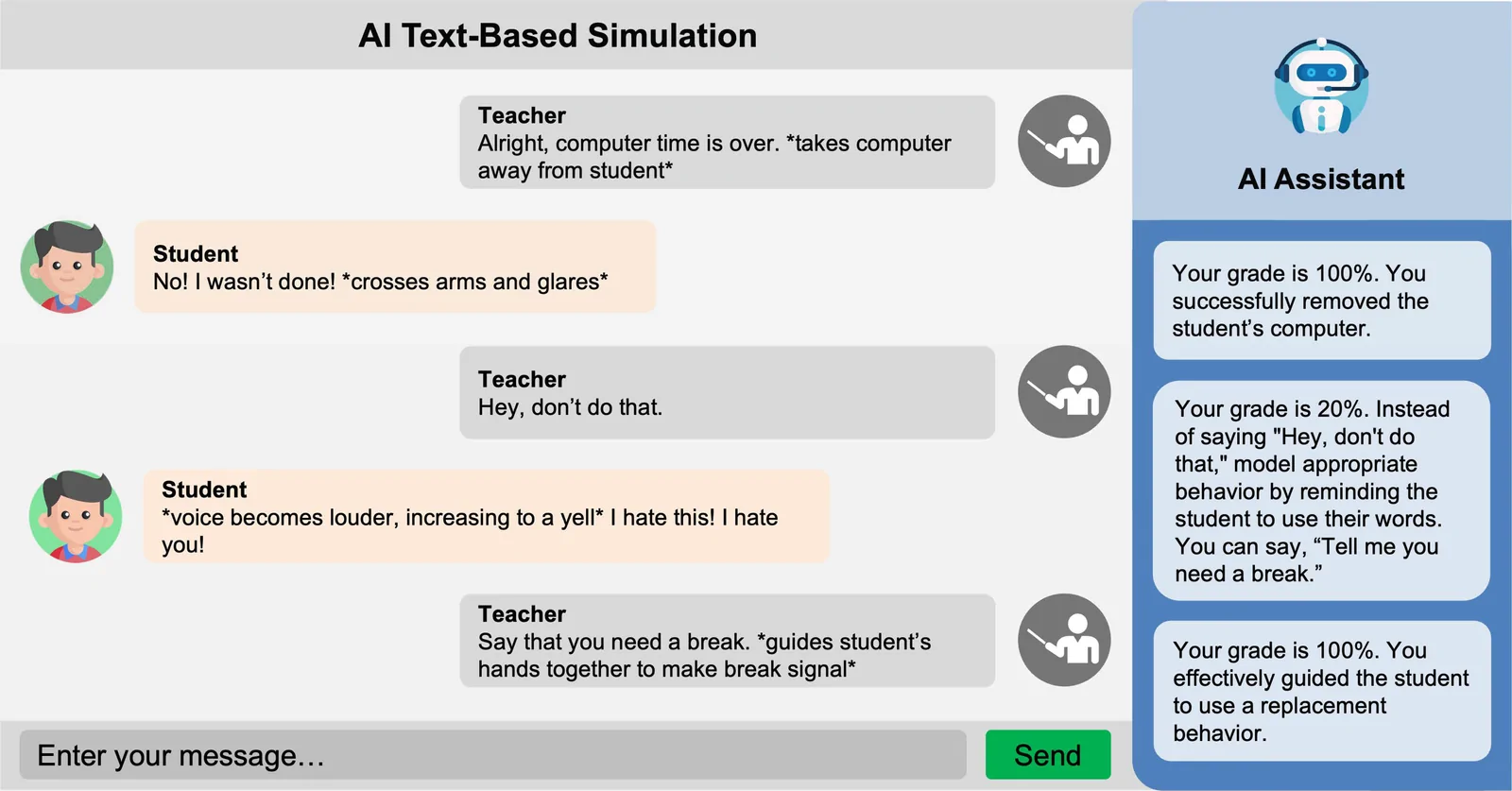
Depiction of interactions within the text-based chatbot simulation during training sessions in an exploratory randomized trial comparing chatbot- and virtual reality (VR)–delivered instruction in functional communication training (FCT). Trainees responded to the simulated student by typing messages in the field at the bottom of the interface. Text bracketed in asterisks denotes described actions accompanying statements.

### Apparatus and Materials

A commercially available desktop, connected to 2 external monitors, was used to project the two 4-minute instructional videos. The monitor view was mirrored to the researchers’ computer, allowing them to confirm playback quality and timing. Chatbot simulations were run on a high-performance Windows laptop connected to a fast campus network, which enabled the experimenters to mirror participants’ screens in real time and verify the automated scoring routines. The laptop was connected to a portable secondary monitor and keyboard, which allowed the principal investigator to monitor trainee performance and maintain control of the chatbot during training sessions.

For the VR condition, participants interacted via standalone Meta Oculus Quest 2 head-mounted displays equipped with native motion controllers. The headsets’ inside-out tracking obviated the need for external sensors, and the built-in microphones and speakers enabled natural voice exchanges with the simulated students. After completing the VR or chatbot sequences, participants used standard Windows desktops to complete project assessments.

### Personnel and Administration

The first author, a faculty member with a PhD in special education and certification as an applied behavior analyst, led all sessions. Four graduate students with backgrounds in special education and school psychology assisted with observations, with no more than one additional observer attending each session. An undergraduate student pursuing a BA in computer science provided technical assistance as needed. All sessions were conducted in English.

### Data Analysis

#### Units of Delivery and Analysis

Simulations were delivered individually, and individual participants were the unit of random assignment.

#### Data Diagnostics

No participants were excluded following enrollment, and no criteria for postenrollment exclusion were applied. We did not anticipate missing data given the scope and administration of the study; however, methods such as imputation would have been selected based on the mechanism responsible for data loss (eg, random, nonrandom). For example, random missingness attributed to momentary technical failure, as encountered in previous studies [19], would be addressed using multiple imputation [48]. Assumptions for the binomial generalized linear mixed model (GLMM; see the Analytic Strategy section), including tests of dispersion, residual uniformity, detection of outliers, and the distribution of random effects, were assessed using the DHARMa package [49] in *R*. Modeling of outcomes as binomial step counts (ie, steps correct out of 18) precluded the need for data transformations.

#### Analytic Strategy

All analyses were conducted in *R* (*α*=.05). FCT implementation performance was analyzed using a binomial GLMM, with a random intercept for participants who contributed repeated OMP and TBP observations in baseline and training sessions. We also modeled fixed effects of training group (chatbot or VR), time (pretest, posttest), probe modality (OMP, TBP), and their interactions. Fixed effects are reported as odds ratios (ORs) with 95% CIs. Simple effects following a significant interaction were estimated as pairwise ORs using estimated marginal means and corrected for multiple comparisons across the 2 posttest contrasts using the Holm adjustment [50]. Given limitations in the interpretation of ORs [51], average marginal effects (AMEs) representing the differences in the proportion of steps completed correctly were also calculated. We did not adjust outcomes by subgroup, and no moderator nor mediator analyses were performed. Models were fit and AMEs calculated using the lme4 [52] and marginaleffects [53] packages.

Differences in pre- and posttest SKIL Survey responses were implemented with an Aligned Rank Transform (ART) ANOVA using the ARTool [54] package. This nonparametric approach enables factorial analysis of ordinal outcomes while preserving the ability to test interaction effects in designs involving both between- and within-subjects factors. The analysis included a within-subjects factor of time (pre, post) and a between-subjects factor of intervention group (VR vs chatbot), with repeated measures nested within participant. We corrected for multiple comparisons across the 9 tests across the 3 subscales using the Holm adjustment [50]. The differences between social validity surveys administered in the final session were analyzed using the Mann-Whitney *U* test, a nonparametric alternative for comparing group differences [55].

## Results

### Participant Flow

Following baseline assessments, the 23 participants were randomly assigned to groups who received either the chatbot (n=11) or VR (n=12) training. No participants withdrew or switched conditions; both groups completed the OMP, TBP, and all secondary measures at follow-up without losses; and all were included in the analysis. A diagram depicting participant flow appears in Figure 3.

**Figure 3.**
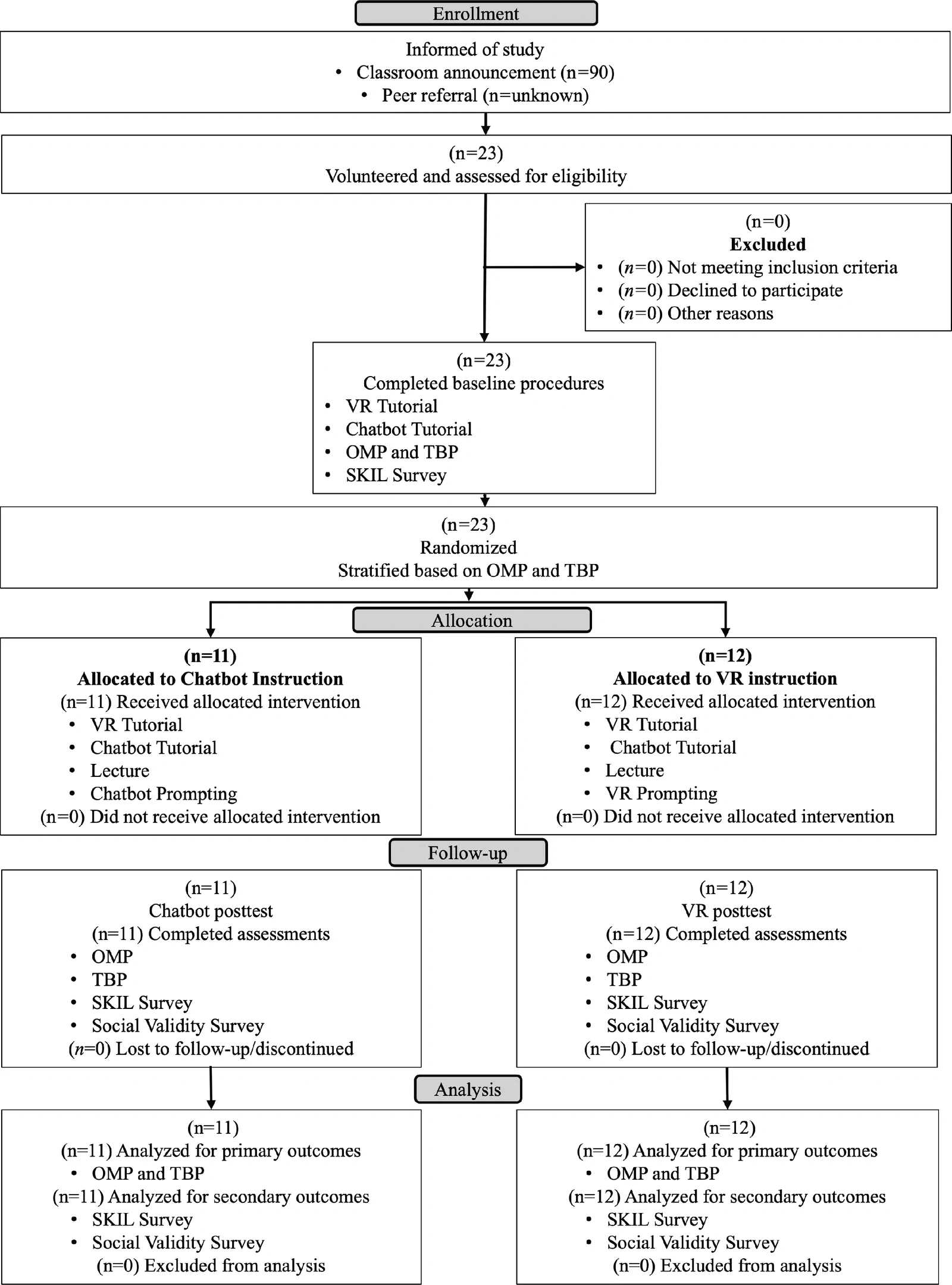
Flow of participants through each stage of an exploratory randomized trial comparing chatbot- and virtual reality (VR)–delivered instruction in functional communication training (FCT) adapted from the CONSORT (Consolidated Standards of Reporting Trials) [56] flow diagram. All participants were included in the analysis. OMP: observed mastery probe; TBP: text-based probe.

### Baseline Equivalence

Groups did not differ significantly on demographic characteristics (Table 1). Descriptive statistics for baseline outcomes, including mean TBP response times, appear in Table 2. Differences between VR and chatbot groups at baseline were not significant for either the OMP (OR 0.89, 95% CI 0.40‐2.01; *P=*.78) or TBP (OR 0.80, 95% CI 0.36‐1.76; *P=*.57).

**Table 1.** Participant demographics by training condition in an exploratory randomized trial of chatbot- and virtual reality (VR)–delivered preparation in functional communication training (FCT) conducted with students recruited from a midwestern university in the United States.

Participant	Total (n=23)	VR^a^ (n=12)	Chatbot (n=11)	Statistical test result (*df*)^b^	*P* value^c^
Age (years), mean (SD), range	24 (5.3), 19-42	25.4 (6.7), 20-42	22.5 (2.8), 19-30	1.37 (15)^d^	.19
Gender, n				0.210 (1)^e^^,^^f^	.65
Male	7	3	4		
Female	15	8	7		
Not disclosed	1	1	0		
Background^g^^,^^h^				0.034 (1)^f^	.86
Education	10	5	5		
General education	8	4	4		
Special education	2	1	1		
Other	13	7	6		
Status				0.217^i^	—^j^
Undergraduate	21	12	9		
Graduate	2	0	2		
Duration in program (years), mean (SD), range				1.242 (19)^d^	.23
Undergraduate	2.8 (1.0), 1-5	3 (1.12), 1-5	2.6 (0.9), 1-4		
Graduate	2 (1.4), 1-3	—	2 (1.4), 1-3		
Education experience (years), n				0.348^i^	—
0	6	4	2		
<1	4	3	1		
1‐4	11	4	7		
5‐10	1	1	0		
>10	1	0	1		
Behavior intervention experience, n				0.379 (1)^f^	.54
Yes	5	2	3		
No/I don’t know	18	10	8		
English proficiency, n				0.491 (1)^f^	.48
First language	20	11	9		
Second language, fluent	3	1	2		
VR experience^k^, mean (SD), range	2.8 (1.4), 1-6	2.6 (1.2), 1-6	3.1 (1.6), 1-6	–0.862 (21)^d^	.40
Voice assistant comfort, mean (SD), range	3.39 (1.5), 1-6	3.58 (1.6), 1-6	3.18 (1.5), 1-6	0.620 (21)^d^	.54

a VR: virtual reality.

b Categorical variables in which expected cell counts <1(ie, status, years in education) were analyzed using Fisher exact tests.

c Differences between groups were not significant at α=.05.

d Independent sample *t* test.

e Comparisons within the gender category included only participants who reported their gender.

f *χ*2 test.

g Participants could select more than one background characteristic.

h Comparisons within the background category only involved “Education” and “Other.”

i Fisher exact test.

j Not applicable.

k Determined using a 6-point Likert-type scale ranging from 1 (no experience) to 6 (much experience).

**Table 2.** Descriptive statistics for baseline and training outcomes by training condition in an exploratory randomized trial of chatbot- and virtual reality (VR)–delivered preparation in functional communication training (FCT).

Variable	Total (n=23)	Chatbot (n=11)	VR (n*=*12)
Baseline			
OMP^a^^,^^b^ , mean (SD), range			
% correct	12.08 (3.61), 11.11‐27.78	11.62 (1.68), 11.11‐16.67	12.50 (4.81), 11.11‐27.78
TBP^c^^,^^d^, mean (SD), range			
% correct	13.28 (4.95), 11.11‐27.78	12.12 (3.35), 11.11‐22.22	14.35 (6.02), 11.11‐27.78
Response time (s)^e^	26.07 (5.52), 13.88‐37.38	26.56 (6.30), 13.88‐37.38	25.61 (4.94), 14.81‐32.44
SKIL variables^f^, mean (SD), range			
Knowledge	2.09 (0.76), 1‐3.78	2.25 (0.75), 1.33‐3.33	1.94 (0.72), 1‐3.78
Confidence	1.95 (0.58), 1‐3.33	1.96 (0.51), 1.33-3	1.94 (0.65), 1‐3.33
Usefulness	2.55 (0.76), 1.22‐3.78	2.55 (0.84), 1.22‐3.67	2.56 (0.72), 1.67‐3.78
Training			
OMP, mean (SD), range			
% correct	75.12 (22.71), 27.78-100	69.70 (25.62), 27.78-100	80.09 (19.46), 55.56-100
TBP, mean (SD), range			
% correct	72.22 (23.03), 27.78-100	82.32 (19.05), 50-100	62.96 (23.13), 27.78‐94.44
Response time (s)	16.17 (2.89), 12.06‐21.57	14.34 (1.54), 12.06‐16.80	17.85 (2.84), 12.86‐21.57
SKIL variables, mean (SD), range			
Knowledge	3 (0.49), 2.00‐3.67	3.15 (0.48), 2‐3.67	2.86 (0.48), 2‐3.67
Confidence	2.95 (0.53), 1.67‐3.67	3.16 (0.33), 2.56‐3.67	2.75 (0.61), 1.67‐3.67
Usefulness	3.44 (0.46), 2-4	3.52 (0.43), 2.78-4	3.37 (0.56), 2-4
Social validity^g^, mean (SD), range			
Acceptability	5.39 (0.66), 3.50‐6.00	5.33 (0.75), 3.50-6	5.45 (0.57), 4.50‐6.00
Effectiveness	5.05 (0.57), 4.00‐6.00	5.08 (0.58), 4‐5.75	5.02 (0.59), 4.00‐6.00
VR	5.09 (1.05), 2.67-6	5.00 (1.16), 2.67‐6.00	5.18 (0.96), 3.67‐6.00
Chatbot	5.07 (0.76), 3.67-6	5.17 (0.61), 4.00‐6.00	4.97 (0.91), 3.67‐6.00

a OMP: observed mastery probe.

b OMP scores represent percentage of steps completed correctly out of 18. OMP was discontinued if response not provided within 4 s or an incorrect response.

c TBP: text-based probe.

d TBP scores represent percentage of steps completed correctly out of 18.

e TBP response duration represents seconds between the simulated student’s message and the trainee’s submitted response. TBP was discontinued after an incorrect response exclusively to permit composition.

f Knowledge, confidence, and usefulness of FCT determined using 4-point scales featured on the SKIL Survey, with 1=none/very low and 4=substantial/very high [38].

g Social validity was assessed across acceptability, effectiveness, and ease of use using a 6-point scale, with 1=strongly disagree and 6=strongly agree.

### Statistics and Analysis

#### Missing Data

All participants completed all sessions and measures. No data were missing on any probe or survey.

#### Assumptions

Model assumptions were evaluated for the binomial GLMM using simulation-based residual diagnostics in DHARMa [49]. These indicated no evidence of overdispersion or underdispersion (dispersion ratio=1.06, *P=*.26) and no outliers. Random effects approximated a normal distribution (Shapiro-Wilk *P=*.10). Although a residual uniformity test indicated a marginal deviation (Kolmogorov-Smirnov *D=*.14, *P*=.04) consistent with bounded, discrete outcomes [49], the absence of dispersion or outliers suggested no substantial violation of model assumptions under binomial models.

#### OMP and TBP Assessments

Descriptive statistics for measures across groups appear in Table 2. A main effect of time (OR 36.09, 95% CI 20.75‐62.75; *P<*.001) indicated significant learning gains for both groups. The 3-way time × probe modality × training group interaction was also significant (OR 6.10, 95% CI 2.08‐17.86; *P<*.001), indicating that the pattern of transfer from pre to post differed by training and probe type. Following the Holm adjustment [50], the chatbot group significantly outperformed the VR group on the TBP (OR 4.14, 95% CI 1.20‐14.24; *P=*.049), completing, on average, approximately 22% more of the steps in the FCT procedure (AME 0.22, 95% CI 0.04‐0.41). On the OMP, differences between groups were not significant (OR 0.61, 95% CI 0.18‐2.07; AME –0.08, 95% CI –0.27 to 0.11; *P=*.43). This pattern of performance appears in Figure 4.

**Figure 4.**
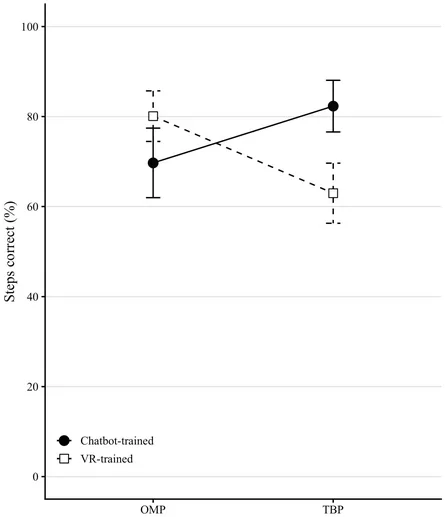
Mean percentage of functional communication training (FCT) steps performed correctly at posttest, by training group and probe modality, in an exploratory randomized trial of chatbot- and virtual reality (VR)–delivered preparation in FCT. On the observed mastery probe (OMP), administered in VR, the 2 groups did not differ significantly; on the text-based probe (TBP), chatbot-trained participants outperformed VR-trained participants. Error bars represent ±1 SE.

#### SKIL Survey

Following the Holm adjustment [50], the ART ANOVA revealed a significant main effect of time for knowledge (*F*_1,21_=26.92, *P*<.001). There was no significant main effect of intervention (*F*_1,21_=2.74, *P*=.68) nor a significant interaction (*F*_1,21_=0.04, *P*>.99). For self-reported confidence, there was a significant main effect of time (*F*_1,21_=36.80, *P*<.001). Neither the main effect of intervention (*F*_1,21_=1.58, *P*>.99) nor the interaction (*F*_1,21_=1.24, *P*>.99) reached significance. For perceived usefulness, there was a significant main effect of time (*F*_1,21_=24.11, *P<*.001). The main effect of intervention (*F*_1,21_=0.03, *P*>.99) and the interaction (*F*_1,21_=0.03, *P*>.99) were not significant.

### Fidelity

Checklists corresponding with appropriate steps for each session were created prior to the beginning of the project and used to assess protocols across all sessions. Specific steps pertained to the use of correct instructions, administration of required assessments, and management of the simulation. All observers were trained by the first author to assess the procedural fidelity prior to the project. Fidelity observers were required to reach the mastery criterion (>90% agreement with the first author) before coding independently. Fidelity observers were required to observe the first author administer 3 sessions and administer a session in the presence of the first author before administering sessions independently. Administrators consulted fidelity checklists during sessions. The research team collected fidelity in baseline (13/23, 57%), VR training (5/12, 42%), and chatbot training (3/11, 27%) sessions. Fidelity was calculated by dividing the number of items on the observation checklist correctly performed by the session administrator by the total number of checklist items. Mean fidelity was 99.70% (SD 1.07%; range 96.15%‐100%) in baseline sessions. Mean fidelity for both VR training and chatbot training sessions was 100%.

### Social Validity

Participants rated the intervention favorably across all domains (Table 2). Mann-Whitney *U* tests indicated no significant differences between the chatbot and VR groups on any domain (*U*=55.50‐62.50, all *P>*.05). On the social validity survey, 10 participants (10/23, 44%) left open‐ended comments. Representative comments, though not formally analyzed, illustrate participant impressions of the training. Several participants noted positive impressions of the VR sessions (eg, “VR session [was] more helpful than chatbot”) but also offered constructive feedback on its mechanics and design (eg, “Eyes in the VR creep me out,” “I had some issues getting the VR system to detect my voice”). A chatbot participant noted that “It was difficult to tell which parts of the screen I should be looking at, or when I needed to try again.” Others praised the video training components (eg, “I liked the diagrams and the video summary”), although some felt the videos were overly dense. One participant expressed reservations about the behavioral strategies, and another felt uneasy rushing through the practice sessions. Despite these concerns, several participants described the project as interesting or beneficial (eg, “This was wild”).

### Adverse Effects

No adverse effects were observed or reported in either condition.

## Discussion

### Principal Findings

This exploratory study compared the effectiveness of 2 aligned multicomponent training packages featuring either VR or chatbot simulations for teaching steps in FCT. Our first question pertained to the improvement participants in each condition exhibited across the OMP and TBP. Although participant performance improved following both training packages, results suggest the pattern of transfer differed by training condition. Whereas participants who trained in the chatbot simulation outperformed VR-trained participants on the TBP, there was no significant difference between the 2 groups on the OMP—yielding a significant 3-way interaction. Notwithstanding the limited power of this study, these findings indicate that the chatbot group’s learning was maintained across both text-based and physical assessments. Our second question pertained to changes in participants’ self-perceived knowledge, confidence, or intervention utility. Across groups, no differences were identified. Participants reported that both modalities were generally acceptable and easy to use. Findings provide tentative support for the use of chatbot training for skills involving speech and movement while underscoring the importance of considering the alignment of training and assessment modality in training evaluation.

This study differs from earlier scholarship concerning VR simulations and chatbots. Few studies have compared immersive simulations with comparison conditions aligned in terms of content and instructional methods [20,21]. The disparity between control conditions in VR studies—which frequently consist of didactic instruction—and immersive learning conditions has previously been justified by the absence of scalable methods of role-play and other activities facilitated through VR [19,57]. That even the modest comparative advantages associated with immersive VR instruction [58] did not materialize in this study is therefore notable given the large advantages of VR simulations identified in our previous scholarship [19]. This likely reflects the nature of the comparison: VR previously outperformed didactic instruction because the latter lacks opportunities for rehearsal, whereas the chatbot condition provided aligned rehearsal of the same procedure.

Previous chatbot instructional literature generally does not pertain to acquisition of procedural skills such as the use of FCT [23]. The within-subject effects demonstrated here suggest that chatbots may be a viable format for procedural rehearsal. As both VR and the chatbot were embedded within the same instructional approach [18], the 2 technologies should be viewed as supplemental to effective instruction rather than the active ingredient [19,20]. The apparent asymmetry in transfer effects between the two modalities, or the extent to which chatbot-trained participants’ performance on OMP did not differ significantly from that of the VR group while outperforming them on the TBP, represents a potential advantage of the chatbot medium that merits closer consideration.

Our findings, which support an advantage of chatbot training for text-based assessment and nonsignificant differences on a VR-based assessment when compared with a group trained using VR, must be interpreted cautiously given the small sample. Leaving this aside, there are additional explanations of the asymmetry in transfer effects. The tutorial included many of the motions needed in the OMP (eg, manipulating objects, grabbing the avatar’s hands). Combined with their text-based training of FCT procedures, this motor experience may have assisted OMP performance. As both groups completed identical VR and chatbot tutorials, their effects were likely constant across conditions. Neither group performed more than a few steps correctly on baseline probes completed after the VR and chatbot tutorials. Results of prior studies similarly suggest the tutorial, in the absence of rehearsal, was not equivalent to the VR training condition [18], even when accompanied by a video explaining the FCT procedure [19]. Given that the tutorial, on its own, was insufficient to produce comparable gains, the chatbot group’s OMP performance in this study is better attributed to the combination of tutorial exposure and text-based FCT rehearsal than to incidental motor practice. The possibility that motor practice divorced from knowledge of the goal—such as the VR tutorial—may support future goal-directed performance has been considered, and rejected, in theoretical work (see [59]). This phenomenon also did not emerge in our previous work [19]. Regardless, tutorials are necessary to separate the challenges of engaging with new technology from effective training [46]. Acclimating learners to any supplemental technology is a critical aspect of equity in service delivery [60]. The extent to which preparing learners for a novel instructional format (ie, VR) unavoidably overlaps with the content is worth considering in future simulation research.

The asymmetry in transfer may also reflect the relative familiarity of the learning channel or the combination of learners’ sensory engagement with content (eg, reading, hearing) and the requirements of any subsequent response (eg, act, write; [61]). These transfer relations do not emerge uniformly and develop as a result of an individual’s instructional history [29-31]. For the chatbot group, transferring skills to the OMP meant generalizing steps acquired through reading and typing (ie, read/type) to a format in which the observation of simulated events—previously rendered in textual terms during the chatbot training—occasioned specific statements or actions (ie, see/say, see/do). This form of transfer is consistent with typical instructional practices [31]. In contrast, VR participants learned to speak or act in response to simulated events and were required by the TBP to read descriptions of events then type a description of the appropriate action. Translating action into description represents a channel most learners have little occasion to develop [31]. In terms of cognitive load, an unfamiliar channel requires learners to allocate resources to deciphering the stimulus–response relation rather than the task itself [35]. Conceptualizing the alignment between instructional and assessment channels in these terms may help developers streamline the demands that instruction and assessment place on learners.

Although responses on the SKIL survey did not differ across conditions, participant perceptions of their knowledge and confidence, as well as the utility of FCT, improved following training. Apart from the asymmetry in skill transfer across modalities, these findings are consistent with the observed improvement in performance and previous research (eg, [18]). Given the dissociation between participant perception and performance, we recommend future work in this area incorporate multiple outcomes [62]. Social validity measures indicated that several aspects of VR (eg, grasping virtual objects via controllers) and the chatbot (eg, orientation of feedback) presented challenges for students. Although technological advances—such as controller-free VR interfaces—may address some of these challenges, an emphasis on user experiences across modalities and diverse user populations is warranted [46].

A discussion of AI-enhanced instructional simulations would be remiss without reference to ethical concerns, including privacy [63] and effective treatment [46]. Reserving technologically advanced, yet functionally inferior, methods for underserved areas could perpetuate poor outcomes for certain groups [64]. Future research must focus on efficacy and the equitability of underlying assessment [46]. Automated systems (eg, speech recognition) typically score performance against local criteria [19]. Standardized procedures for establishing the validity of automated assessment would permit meaningful comparisons across studies and increase the generality of findings [65].

### Limitations

This study has several limitations. The small sample in this underpowered exploratory study reduced our ability to detect small to moderate effects. Thus, the absence of significant effects between VR and chatbot groups on the OMP may be a statistical artifact rather than an indication of the equivalence of the two formats. Significant effects were estimated with wide confidence intervals that warrant replication. Future studies should analyze multiple factors in the context of adequately powered comparisons of modally distinct but fundamentally aligned instruction [66]. Observers responsible for scoring probes were aware of participant training conditions, which introduces a risk of expectancy bias. Additionally, the sample consisted of a relatively homogeneous group of college students, some of whom studied topics with little relation to FCT. Future studies should incorporate broader and more diverse participant samples, including in-service professionals and individuals from varied educational or clinical settings, as well as those from culturally and linguistically diverse backgrounds whose experience or attitudes toward technology may differentially affect results.

Although probes were functionally aligned, there were differences in procedures. The most salient of these is the lack of a time-based discontinuation rule for the TBP. This difference was a property of the probe rather than training group: All participants, regardless of group, completed both the OMP and TBP. The advantage of chatbot training on the TBP cannot be attributed to the timing asymmetry exclusively, as both groups completed the probe under identical conditions. Mean response time on the posttest TBP (Table 2), which indicated that the VR group took more time to complete responses despite their lower scores, suggests the VR group did not benefit from the absence of the time constraint on the TBP. The chatbot training sessions, including assessments and tutorials, were approximately 10 minutes longer than the VR sessions. This may have resulted in a difference in engagement time, yet this does not appear to have led the chatbot group to outperform the VR group on both probes. Research interested in standalone measures of performance should align administration parameters to the greatest extent possible and validate the results against applied performance. This study did not evaluate in vivo generalization. Future research should examine how skills acquired through VR or chatbot interactions transfer to naturalistic or live settings, such as classrooms, community-based environments, or homes.

### Future Directions

This study compared the effectiveness of two emerging approaches to procedural training. Results are tentative and may be attributed to methodological factors. Future work might also manipulate the alignment between training and assessment channels directly, testing whether the transfer asymmetry observed here reflects the relative familiarity of the response channels each modality requires. The extent to which aligned instruction produces differential outcomes across specific training modalities such as high-fidelity or virtual simulations [66]—and efforts to facilitate the development and alignment of evaluation platforms—represents an important area for further study [21]. A related area concerns the use of technologies such as AI-augmented VR and chatbots within more authentic contexts (eg, coursework), as opposed to isolated simulations [67]. At minimum, the study provides support for the use of chatbots in the acquisition of procedural skills, which may serve to support ongoing work examining the incorporation of chatbots into various instructional settings as well as their interplay with other technology platforms [68].

The ultimate utility of instructional simulations is determined by the extent to which they influence practice. The same features that necessitate automated simulations for skills such as FCT—risk for injury and fluidity of clinical situations [13,19]—also impede efforts to evaluate effects of instruction on professional practice and client outcomes. Validating the instructional model on lower-risk skills amenable to this approach may be warranted.

### Conclusions

This study—to our best knowledge, the first comparison of aligned chatbot- and VR-based FCT training simulations—suggests that multicomponent trainings featuring either chatbot- or VR-delivered rehearsal improved participants’ acquisition of a complex educational procedure, with similar improvements in participant knowledge, confidence, and perceived usefulness. Whereas many studies compare VR to didactic instruction or assess performance within a single modality, this study compared VR with a chatbot capable of providing comparable opportunities for rehearsal. Findings contribute to the field by supporting the viability of chatbots for procedural training and by increasing the salience of alignment between modalities emphasized in training and assessment. Positive findings for chatbots are particularly encouraging given the potentially lower costs of development and deployment relative to VR, which could increase access to effective practices in behavior intervention and related fields. Additional work with larger samples is needed to confirm the observed transfer asymmetry and to examine the effects of these approaches on practitioners in applied settings.

## Supplementary material

10.2196/105569 Multimedia Appendix 1 Description of chatbot system prompts.

## Abbreviations

AI: artificial intelligence
AME: average marginal effect
ART: Aligned Rank Transform
CONSORT-EHEALTH: Consolidated Standards of Reporting Trials of Electronic and Mobile Health Applications and Online Telehealth
FCT: functional communication training
GLMM: generalized linear mixed model
IDD: intellectual and developmental disabilities
IOA: interobserver agreement
IRB: institutional review board
LLM: large language model
OMP: observed mastery probe
OR: odds ratio
TBP: text-based probe
VR: virtual reality
